# Cardioprotective effects of garcinol following myocardial infarction in rats with isoproterenol-induced heart failure

**DOI:** 10.1186/s13568-020-01065-9

**Published:** 2020-08-04

**Authors:** Man Li, Xuewen Li, Lifeng Yang

**Affiliations:** Department of Cardiology, Shanxi Bethune Hospital (Shanxi Academy of Medical Sciences), NO. 99 Longcheng Street, Taiyuan, Shanxi 030000 China

**Keywords:** Garcinol, Digitalis, Apoptosis, Interstitial fibrosis, H9C2 cells

## Abstract

Myocardial infarction is a clinical form of necrosis in the myocardium caused by an imbalance between the coronary blood supply and myocardial demand. Garcinol is a polyisoprenylated benzophenone found in the fruit of *Garcinia indica*, which is abundant in tropical regions. This fruit contains high levels of garcinol, isoxanthochymol, isogarcinol, hydroxycitric acid and xanthochymol. Garcinol and hydroxycitric acid have been shown to have antioxidant effects. In this study, rats were assigned to sham, control, low-dose, high-dose and positive control groups. Hemodynamic and apoptotic markers were evaluated, and histopathological analysis was conducted. The mRNA and protein levels of caspase-3, Bax, Bcl-2 and cleaved caspase-3 were quantified. Garcinol treatment increased the heart rate and improved the maximum rate of increase in left-ventricle (LV) pressure (+d*p*/d*t*_max_), maximum rate of decrease in LV pressure (–d*p*/d*t*_max_), LV ejection fraction and LV systolic pressure in rats with induced heart failure. Garcinol treatment reversed body, liver and heart weight changes, resulting in returns to near-normal levels. In the garcinol treatment group, the number of broken fibers, extent of inflammatory cell infiltration and rate of apoptosis remained within normal ranges. Garcinol reduced the cross-sectional areas of cardiomyocytes, and reduced interstitial fibrosis to a normal level. The mRNA and protein levels of cleaved caspase-3, caspase-3 and Bax were reduced, whereas those of Bcl-2 were increased, following high-dose (100 mg/kg) garcinol treatment. These findings suggest that garcinol effectively prevents apoptosis in rats with isoproterenol-induced heart failure and in cardiac H9C2 cells.

## Introduction

Myocardial infarction is a clinical form of necrosis in the myocardium caused by an imbalance between the coronary blood supply and myocardial demand (Srikanth et al. 2009). Increased free radical production occurs under ischemic conditions (Lord et al. 2011), and reactive oxygen species play central roles in several cardiac and metabolic disorders (Panth et al. 2016). Isoproterenol is a synthetic catecholamine that induces stress in the myocardial muscles (Fan 2019). It has been shown to increase the rates of free radical production and lipid peroxidation, which leads to further necrosis in myocardial muscles (Khalil et al. 2015). Zhang et al. (2008) reported that the damage to myocardial tissue caused by isoproterenol is irreversible.

The plant flavonoids and phenolic compounds provide defense against oxidative stress (Amjad and Shafighi 2013). Flavonoids act as excellent antioxidants due to their free radical scavenging activity and protect tissue against free radical mediated lipid peroxidation (Knekt et al. 2002). Shah et al. (2019) evaluated the cardioprotective effects of several plant-derived products. Garcinol is a well-known polyisoprenylated benzophenone found in the fruit of *Garcinia indica*, which is abundant in tropical regions (Zhao et al. 2018). This fruit contains high concentrations of garcinol, isoxanthochymol, isogarcinol, hydroxycitric acid and xanthochymol (Chattopadhyay and Kumar 2006). Garcinol and hydroxycitric acid are known to have antioxidant properties (Liao et al. 2005). Patel et al. (2015) reported the cardioprotective potential of *Garcinia indica* extract, based on a rat model of isoprenaline-induced myocardial injury. Garcinol is a good antioxidant present in *Garcinia indica* which has structural similarity to curcumin as it contains both phenolic hydroxyl groups and a β-diketone moiety (Singh et al. 2011). Sutar et al. (2012) have reported the free radical scavenging activity of garcinol. Various plant-derived compounds have been shown to have cardioprotective potential through antioxidant effects (Haleagrahara et al. 2011; Fathiazad et al. 2012). During heart failure, cardiomyocyte apoptosis leads to cardiomyopathy (Li et al. 2014). We hypothesized that garcinol would exhibit protective potential in rats with isoproterenol-induced heart failure. This study was conducted to evaluate the cardioprotective effect of garcinol following myocardial infarction in rats with isoproterenol-induced heart failure.

## Materials and methods

### Materials

Garcinol (G5173), isoproterenol (I6504), bovine serum albumin, Dulbecco’s modified Eagle medium, dimethyl sulfoxide (DMSO) and fetal bovine serum were obtained from Sigma-Aldrich (Shanghai, China). H9C2 cardiac cells were obtained from the American Type Culture Collection (USA).

### Animals

Male albino Wistar rats (180–210 g) were obtained from the Animal House of Shanxi Bethune Hospital, NO. 99Longcheng Street, Taiyuan 030000, Shanxi, China. They were kept in standard rat cages under a 12/12-h light/dark cycle and standard atmospheric conditions (60% ± 5% relative humidity at 25 °C ± 0.5 °C). The rats were provided access to water and food ad libitum.

### Animal model

Isoproterenol was used to induce heart failure in the rats. It was administered at 5 mg/kg for 7 consecutive days (Jing et al. 2015).

### Experimental groups

The rats were assigned to sham, control, high-dose (100 mg/kg garcinol), low-dose (10 mg/kg garcinol) and positive control (0.0225 mg/kg digitalis) groups. Garcinol and digitalis were dissolved in DMSO, and the respective doses (total volume, 0.5 mL) were administered orally for 30 consecutive days. H9C2 cardiac cells were separated into normal control, control, high-dose (100 mg/L digitalis), low-dose (10 mg/L digitalis) and positive control (0.0225 mg/L digitalis) groups. The cells were treated for 48 h.

### Evaluation of hemodynamic markers and histopathological analysis

Hemodynamic parameters were assessed according to Yuan et al. (2012). The rats’ heart rate (HR), left ventricular systolic pressure (LVSP), left ventricular ejection fraction (LVEF), maximum rate of increase in left ventricular (LV) pressure (+d*p*/d*t*_max_) and maximum rate of decrease in LV pressure (−d*p*/d*t*_max_) were measured.

Histopathological examination of heart tissues was performed according to Molh et al. (2008). The mounted heart tissue specimen was observed and was scored under light microscopy. For a semi-quantitative comparison of the structural changes, the abnormalities in the tissue sections were graded from 0% (normal structure) to 100% (severe pathological changes).

### TUNEL assay

Terminal deoxynucleotidyl transferase dUTP nick end labeling (TUNEL) assays were conducted according to a previously described method (de Torres et al. 1997). The numbers of apoptotic cells in rat heart tissue and apoptotic cardiac H9C2 cells were determined in six randomly selected samples each. Analysis of fluorescence intensity through ImageJ was described in the documentation section of ImageJ and can be found at the URL: https://imagej.nih.gov/ij/docs/.

### Rt-pcr

Total RNA was isolated from heart tissue homogenate and converted into cDNA. The mRNA levels of Bax, caspase-3 and Bcl-2 were quantified using the 2^−∆∆*C*T^ method (Yu et al. 2015). The primers used for mRNA amplification are listed in Table 1.

**Table 1:** List of primers used for the mRNA amplification of caspase-3, Bax, and Bcl-2

Markers	Sense primer	Anti-sense primer
GAPDH	5′-TCCCTCAAGATTGTCAGCAA-3′	5′-AGATCCACAACGGATACATT-3′
Bax	5′-TGG AGCTGCAGAGGATGATTG-3′	5′-GAAGTTGCCGTCAGAAAACATG-3′
Caspase-3	5′-TTAATAAAGGTATCCATGGAGAACACT-3′	5′-TTAGTGATAAAAATAGAGTTCTTTTGTGAG-3′
Bcl-2	5′-CAC CCC TGG CAT CTT CTC CTT-3′	5′-AGC GTC TTC AGA GAC AGC CAG-3′

### Western blot analysis

Protein levels in heart tissue homogenate were measured. After the isolation of proteins in the lysate, non-specific proteins were blocked using 5% non-fat milk powder. The membranes were incubated with primary antibodies for Bax (1:500 dilutions), caspase-3 (1:300 dilutions) and Bcl-2 (1:500 dilutions) for 12 h, then washed carefully and treated with horseradish peroxidase–conjugated IgG antibodies for 60 min. Protein levels of Bax, caspase-3 and Bcl-2 were determined using western blot analysis (Zhang et al. 2012).

### Statistical analysis

Values are reported as means ± standard deviations. Analysis of variance was applied to the data, and Tukey’s post hoc tests were used for comparisons. *P* values < 0.05 were considered to be significant.

## Results

In this study, the effects of garcinol in rats with isoproterenol-induced heart failure were investigated. Hemodynamic markers were measured after 30 days of garcinol administration. Garcinol treatment improved the LV +d*p*/d*t*_max_, LV –d*p*/d*t*_max_, LVEF and LVSP and increased the HR (all *P *< 0.05; Table 2). Body, liver and heart weight changes differed between control and treated rats (all *P *< 0.05; Table 3). Garcinol treatment reversed the weight changes, leading to returns to near-normal levels.

**Table 2:** Effects of garcinol on hemodynamic markers and cardiac function in isoproterenol**-**treated rats

Hemodynamic markers	Sham	Control	Low-dose	High-dose	Digitalis
SBP (mmHg)	99.2 ± 10.1	72.5 ± 6.5*	77.8 ± 5.1	92.4 ± 9.1^#^	96.5 ± 8.2^##^
DBP (mmHg)	59.1 ± 5.2	44.3 ± 4.1*	46.2 ± 4.2	52.5 ± 4.1^#^	54.1 ± 4.3^#^
HR (beat/min)	358.2 ± 16.2	296.1 ± 12.2*	317.2 ± 14.2	351.3 ± 14.1^#^	355.2 ± 15.4^##^
+LV d*p*/d*t*_max_ (mmHg/s)	7226.5 ± 681.3	3145.1 ± 273.2***	3818.2 ± 319.2^#^	6915.2 ± 510.2^##^	6716.3 ± 562.5^###^
−LV d*p*/d*t*_max_ (mmHg/s)	5581.5 ± 511.3	2811.4 ± 191.2***	3443.1 ± 241.5^#^	5412.2 ± 291.3^##^	5113.2 ± 325.6^###^
LVSP	126.5 ± 11.5	77.3 ± 5.1*	91.4 ± 4.6^#^	119.8 ± 7.1^##^	117.2 ± 8.1^###^
LVEF	75.3 ± 5.1	50.7 ± 4.1*	59.4 ± 3.8	72.2 ± 4.8^#^	69.5 ± 4.2^#^

±LV dp/dtmax: Maximal rates of increase and decrease of left-ventricle pressure development. Data are expressed as mean ± SEM. *n*= 6 in each group

*HR* Heart rate, *DBP* Diastolic blood pressure, *SBP* Systolic blood pressure, *LVSP* Left ventricular systolic pressure, *LVEF* Left ventricular ejection fraction

*P< 0.05 ***vs*** group I

^#^*P*< 0.05

^##^*P*< 0.01

^###^*P*< 0.001 vs group II

**Table 3:** Effects of garcinol on heart and liver weight in isoproterenol-treated rats

Treatment	Body weight (g)	Heart (g)	Liver (g)
Sham	197.5 ± 5.5	0.62 ± 0.10	8.6 ± 0.2
Control	162.2 ± 6.2*	0.74 ± 0.07*	12.1 ± 0.2*
Low-dose	171.3 ± 5.1	0.71 ± 0.05	11.5 ± 0.2
High-dose	185.2 ± 5.4^#^	0.65 ± 0.04	9.4 ± 0.2^#^
Digitalis	194.3 ± 6.1^#^	0.66 ± 0.07^#^	10.2 ± 0.2^#^

Data are expressed as mean ± standard deviations. *n *= 6 in each group

^*^P < 0.05 *vs* group I

^#^*P *< 0.05 *vs* group II

**Fig. 1:**
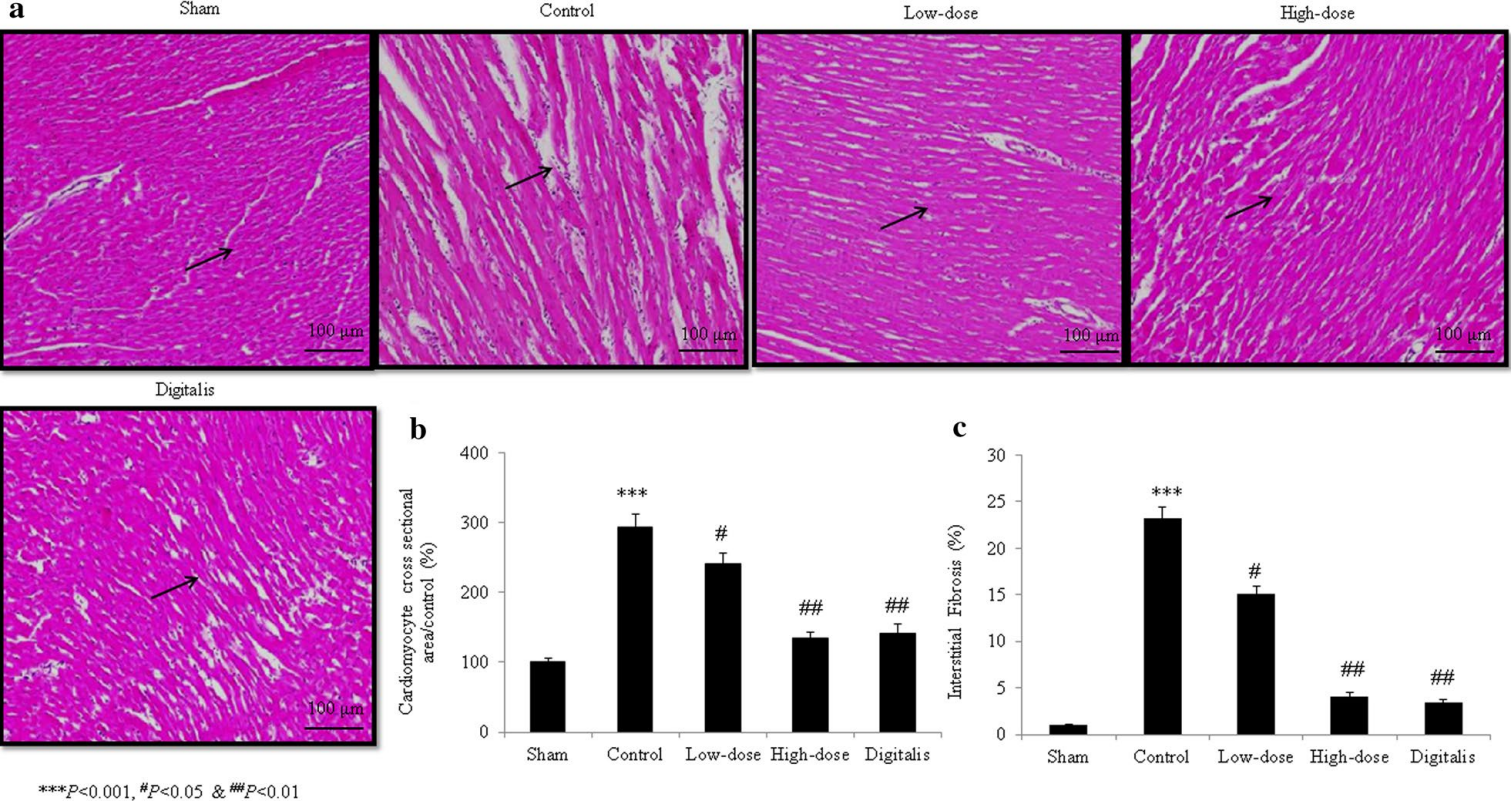
Histopathological evidence of the protective effects of garcinol in heart tissue from rats with isoproterenol-induced heart failure. **a** Microscopic view of rat heart tissue stained with hematoxylin and eosin. **b** Cardiomyocyte cross-sectional area. **c** Interstitial fibrosis. ^#^*P *< 0.05, ^##^*P *< 0.01, ^***^P < 0.001. Scale bar = 100 μm

**Fig. 2:**
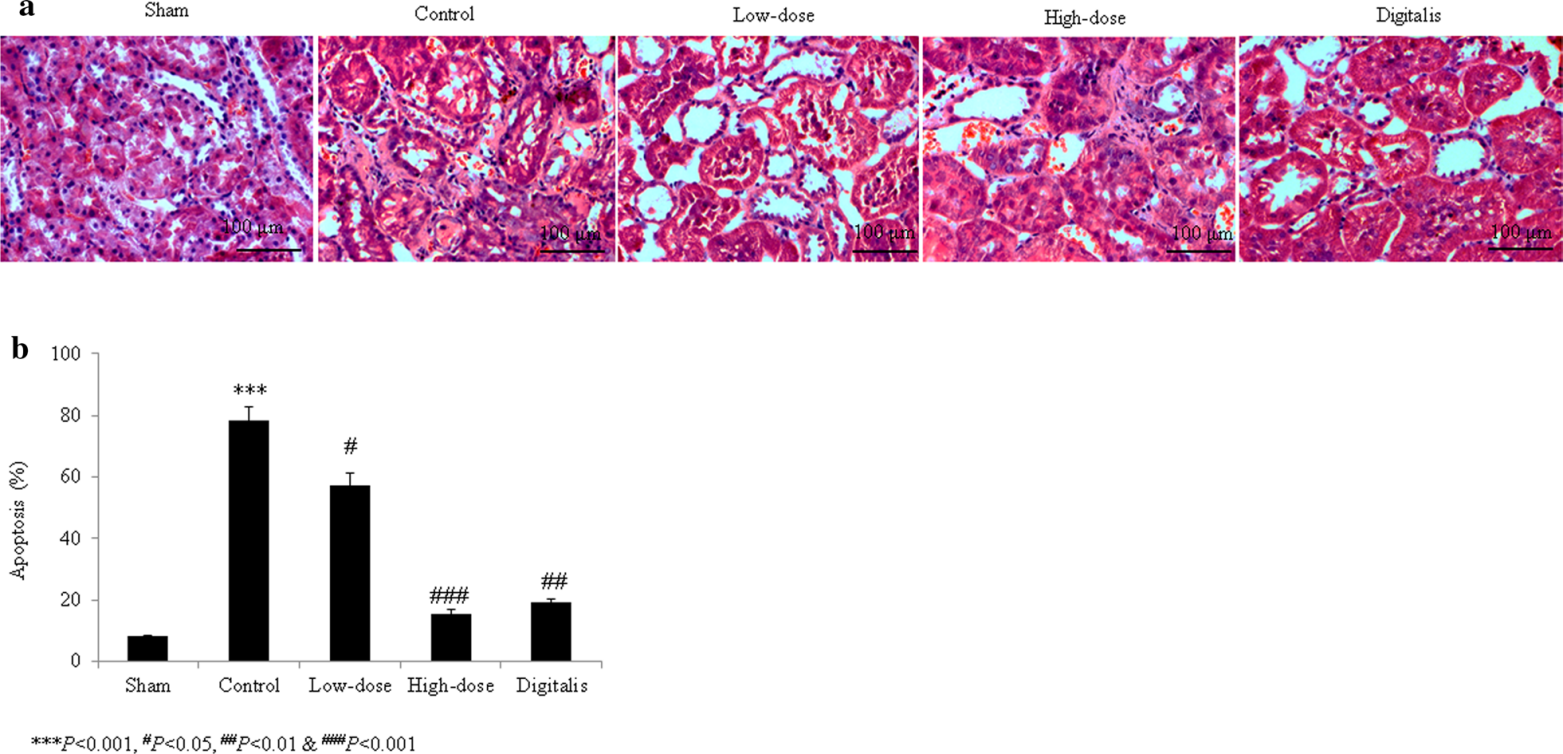
Terminal transferase-mediated dUTP nick end labeling (TUNEL) assay results indicating the protective effects of garcinol against apoptosis in heart tissue from rats with isoproterenol-induced heart failure. **a** TUNEL images of rat heart tissue. **b** Percentages derived from (**a)**. ^#^*P *< 0.05, ^##^*P *< 0.01, ^###^*P *< 0.001, ^***^P < 0.001. Scale bar = 100 μm

**Fig. 3:**
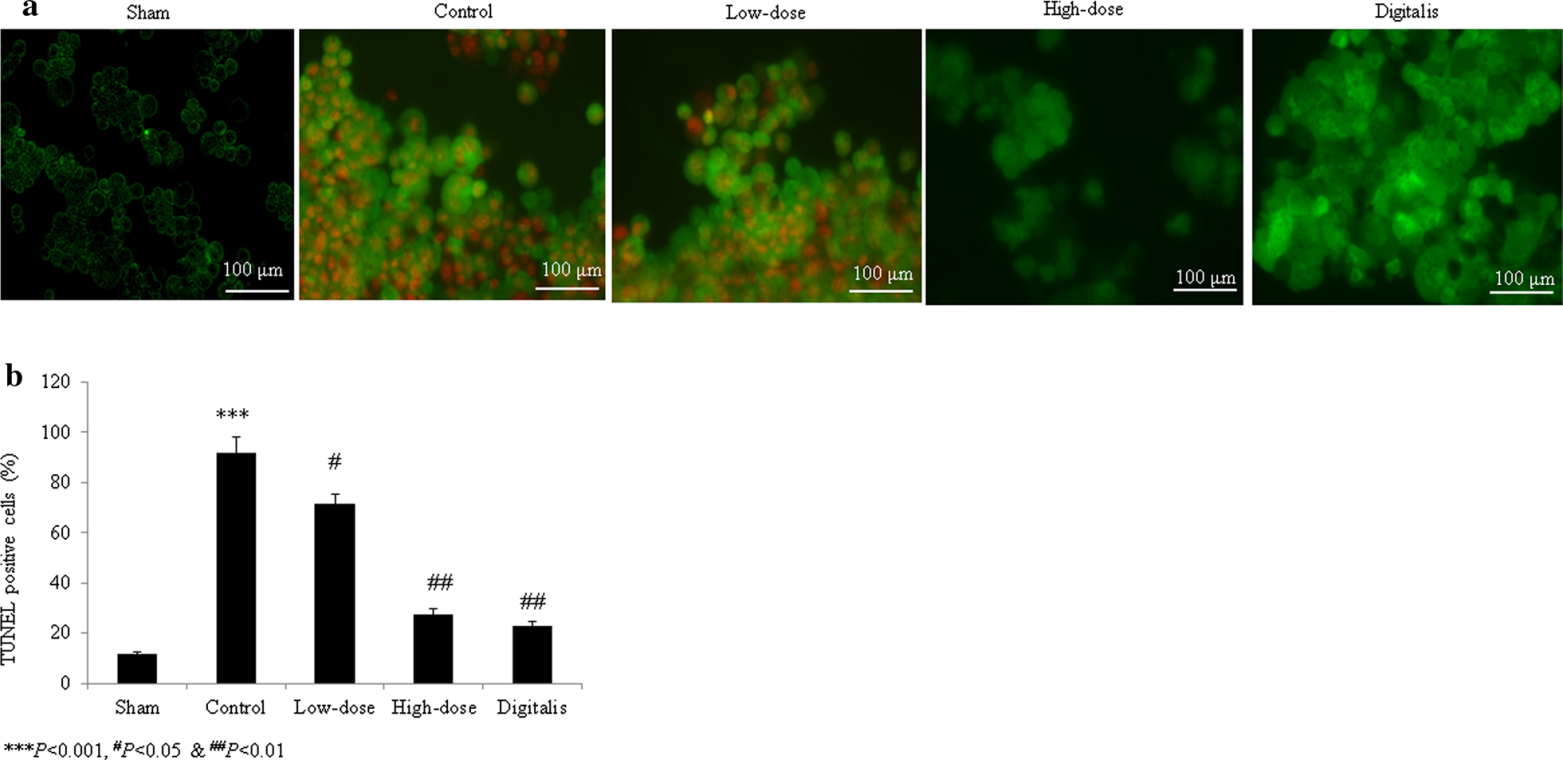
Terminal transferase-mediated dUTP nick end labeling (TUNEL) assay results indicating the protective effects of garcinol against apoptosis in isoproterenol-treated cardiac H9C2 cells. **a** TUNEL images of cardiac H9C2 cells. **b** Percentages derived from (**a)**. ^#^*P *< 0.05, ^##^*P *< 0.01, ^###^*P *< 0.001, ^***^P < 0.001. Scale bar = 100 μm

Sham-operated rats had normal myocardial cell nuclei and no broken myocardial fibers. A greater degree of inflammatory cell infiltration and broken myocardial fibers were observed in control rats. In the garcinol-treated and positive control groups, the degrees of inflammatory cell infiltration and numbers of broken myocardial fibers remained within the normal ranges (Fig. 1). The cardiomyocytes of control rats had increased cross-sectional areas, and garcinol treatment reduced these areas (*P *< 0.05; Fig. 1b). The extent of interstitial fibrosis was greater in control rats and lesser in the garcinol- and digitalis-treated groups (all *P *< 0.05; Fig. 1c). More apoptosis was observed in control H9C2 cardiac cells and control rats than in cells and rats treated with garcinol and digitalis (all *P *< 0.05; Figs. 2a, b, 3a, b).

**Fig. 4:**
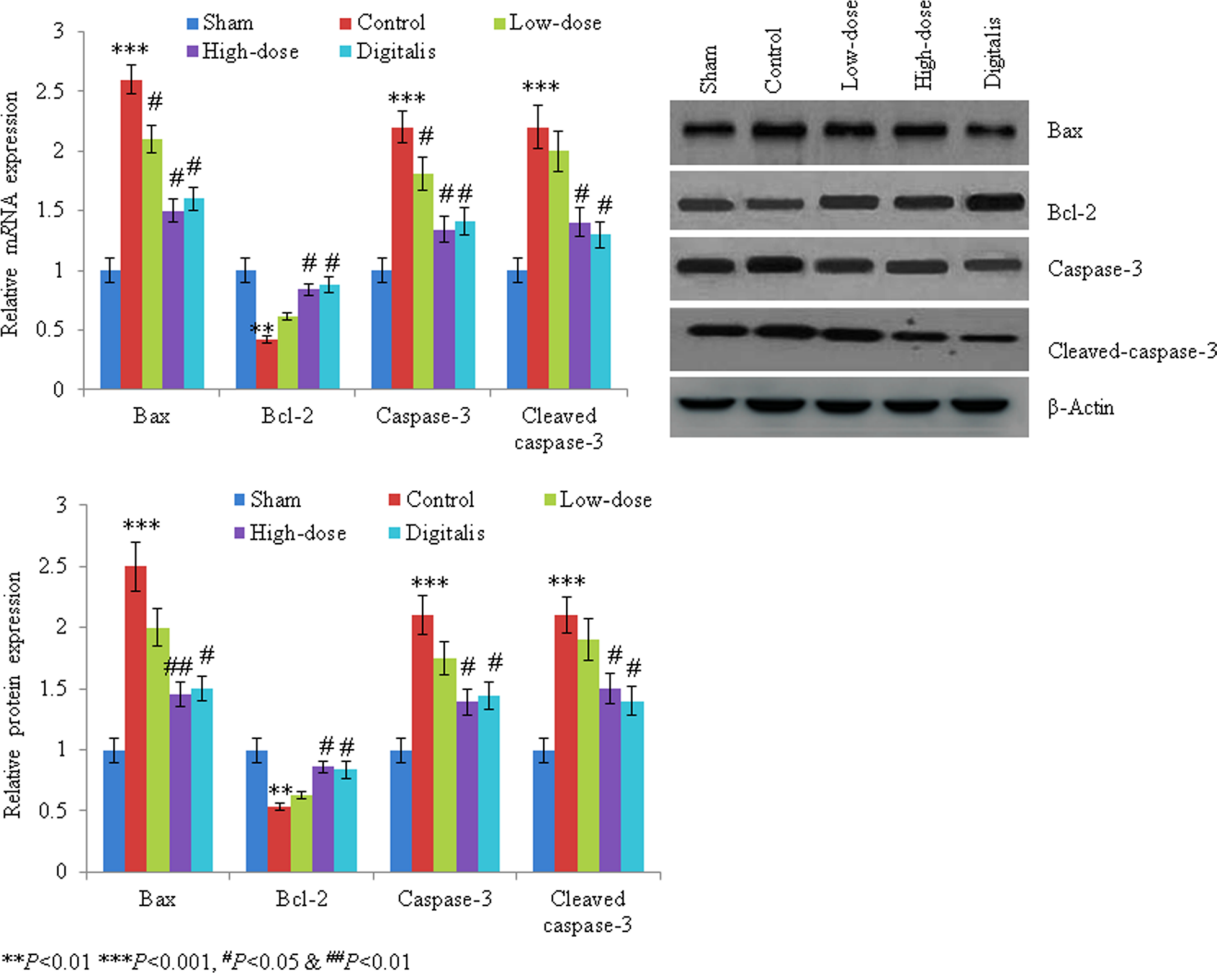
mRNA expression (**a**), western blotting (**b**) and protein expression (**c**) of caspase-3, Bax, Bcl-2 and cleaved caspase-3. ^*^P < 0.05, ^#^*P *< 0.05, ^##^*P *< 0.01, ^***^P < 0.001

RT-PCR was used to quantify the mRNA levels of Bax, caspase-3, Bacl-2 and cleaved caspase-3. In the control rats, mRNA levels of Bax, caspase-3 and cleaved caspase-3 were increased by 160%, 120% and 120%, respectively, whereas Bcl-2 mRNA expression was reduced by 58% (all *P *< 0.05; Fig. 4a). Garcinol treatment reversed these effects, leading to returns to near-normal mRNA levels. Western blotting showed increased protein levels of Bax (150%), caspase-3 (110%) and cleaved caspase-3 (110%), and decreased Bcl-2 mRNA expression (47%) in control rats (all *P* < 0.05; Fig. 4b, c). Garcinol treatment reversed these changes, leading to returns to near-normal protein levels (all *P *< 0.05; Fig. 4b, c).

## Discussion

This study investigated the therapeutic efficacy of garcinol in rats with isoproterenol-induced heart failure and cardiac H9C2 cells. Thanachartwet et al. (2016) described the key roles of several hemodynamic markers in cardiac function. The LV end diastolic pressure and -–d*p*/d*t*_max_ reflect the degree of myocardial relaxation, whereas the LVSP and +d*p*/dt_max_ reflect the degree of myocardial contraction (Wang et al. 2017). In this study, garcinol treatment significantly increased the HR, LVSP, systolic blood pressure and diastolic blood pressure, and reduced the LV –d*p*/d*t*_max_. The high dose of garcinol (100 mg/kg) and positive control treatment (0.0225 mg/kg digitalis) showed greater protective potential than did the low dose of garcinol (10 mg/kg), suggesting that garcinol had a positive inotropic effect in rats with isoproterenol-induced heart failure.

Cardiovascular risk factors, such as inflammation, dyslipidemia, metabolic syndrome and hypertension, are more prevalent in obese and overweight individuals (Lavie et al. 2013; Archer et al. 2013). Changes in body, liver and heart weight were measured in control and treated rats in this study. The garcinol treatment reversed such changes, restoring these weights to near-normal levels, suggesting that it can protect against excess weight gain. Histological examination of myocardial tissue is conducted to diagnose heart failure (Inamdar and Inamdar 2016). In this study, the occurrence of degenerative myocardial vacuoles, disordered arrangement of myocardial cells, fractured myocardial fibers, apoptosis and inflammatory cell infiltration was reduced significantly following garcinol treatment. These findings suggest that garcinol has protective effects on myocardial cells.

Shah et al. (2019) evaluated the cardioprotective effects of several plant-derived compounds. The fruit of the *Garcinia indica* plant is known to contain high levels of garcinol, isoxanthochymol, isogarcinol, hydroxycitric acid and xanthochymol (Chattopadhyay and Kumar 2006). Garcinol and hydroxycitric acid have been shown to have antioxidant effects (Liao et al. 2005). Patel et al. (2015) observed that *Garcinia indica* extract had cardioprotective potential in a rat model of isoprenaline-induced myocardial injury. In myocardial injury, caspase proteins are involved in the cascade leading to apoptosis (McIlwain et al. 2013; Snigdha et al. 2012). These results show that caspase-3 plays an active role in apoptosis regulation in garcinol-treated rats.

In this study, garcinol treatment decreased the levels of cleaved caspase-3 and caspase-3, indicating its inhibitory effect on apoptosis, in rats with isoproterenol-induced heart failure. Nakamura et al. (2000) reported that Bax/Bcl-2 played an active role in the apoptosis cascade in cardiomyocytes. In this study, the protein expression of Bax was decreased and that of Bcl-2 was increased in heart tissue following garcinol treatment. Taken together, these findings suggest that garcinol protects against apoptosis in rats with isoproterenol-induced heart failure and in cardiac H9C2 cells.

## Data Availability

Corresponding author could provide the all experimental data on valid request.
